# A Preliminary Study of Chemical Profiles of Honey, Cerumen, and Propolis of the African Stingless Bee *Meliponula ferruginea*

**DOI:** 10.3390/foods10050997

**Published:** 2021-05-02

**Authors:** Milena Popova, Dessislava Gerginova, Boryana Trusheva, Svetlana Simova, Alfred Ngenge Tamfu, Ozgur Ceylan, Kerry Clark, Vassya Bankova

**Affiliations:** 1 Institute of Organic Chemistry with Centre of Phytochemistry, Bulgarian Academy of Sciences, 1113 Sofia, Bulgaria; popova@orgchm.bas.bg (M.P.); dessislava.gerginova@orgchm.bas.bg (D.G.); bobi_tru@orgchm.bas.bg (B.T.); svetlana.simova@orgchm.bas.bg (S.S.); 2 Department of Chemical Engineering, School of Chemical Engineering and Mineral Industries, University of Ngaoundere, 454 Ngaoundere, Cameroon; macntamfu@yahoo.co.uk; 3 Food Quality Control and Analysis Program, Ula Ali Kocman Vocational School, Mugla Sitki Kocman University, 48147 Ula Mugla, Turkey; ozgurceylan@mu.edu.tr; 4 Volunteer Advisor in Beekeeping and Bee Products with Canadian Executive Services Organization, P.O. Box 2090, Dawson Creek, BC V1G 4K8, Canada; kccsclark@gmail.com

**Keywords:** stingless bees, African Meliponini, pot-honey, propolis, cerumen, chemical profiling, antimicrobial, anti-quorum sensing

## Abstract

Recently, the honey and propolis of stingless bees have been attracting growing attention because of their health-promoting properties. However, studies on these products of African Meliponini are still very scarce. In this preliminary study, we analyzed the chemical composition of honey, two cerumen, and two resin deposits (propolis) samples of *Meliponula ferruginea* from Tanzania. The honey of *M. ferruginea* was profiled by NMR and indicated different long-term stability from *Apis mellifera* European (Bulgarian) honey. It differed significantly in sugar and organic acids content and had a very high amount of the disaccharide trehalulose, known for its bioactivities. We suggested trehalulose to be a potential marker for African stingless bee honey analogously to the recent proposal for Meliponini honey from Asia, South America, and Australia and demonstrated its easy discrimination by ^13^C NMR. Propolis and cerumen were studied by GC-MS (gas chromatography-mass spectometry). The samples contained mainly terpenoids (di-and triterpenes) but demonstrated qualitative and quantitative differences. This fact was an indication that possibly *M. ferruginea* has no strict preferences for resins used to construct and protect their nests. The antimicrobial and anti-quorum sensing properties of the two materials were also tested. These first results demonstrated that the honey, cerumen, and propolis of African stingless bees were rich in biologically active substances and deserved further research.

## 1. Introduction

Stingless bees (Meliponini, Apidae) are closely related to honeybees *Apis mellifera,* but their stings are reduced and not used for defense, thus the name stingless. The Meliponini live in the tropical regions of the world: South and Central America, Southeast Asia, tropical Africa, and Australia [1]. Like honeybees, they are eusocial and produce honey, pollen, wax, and propolis/cerumen [2]. Except for food, their products have been used in traditional medicine for centuries, and recent studies revealed their pharmaceutical potential [3,4]. Over the last years, the interest in stingless bees and their products has been steadily growing [5]. However, studies on African Meliponini are still comparatively rare, and this is particularly true for their honey and propolis [6]. In this article, we described, for the first time, the chemical profiling of honey, propolis (resin loads), and cerumen (the material used by stingless bees for nest construction has sometimes been called batumen. However, according to Simone-Finstrom and Spivak [7], when resins are amalgamated with soil or clay material, the resulting mixture is called geopropolis or batumen, whereas, when it is only mixed with wax, it is called cerumen, with respect to non-honey-bee species). In the case of *M. ferruginea*, no soil/clay was detected, thus, we use cerumen) of the African stingless bee *Meliponula* (*Axestotrigona*) *ferruginea* (Lepeletier) from the region of Kilimanjaro, Tanzania. *M. ferruginea* belong to the genus *Meliponula*, subgenus *Axestotrigona*. The surrounding of the nest entrance of these bees is smeared with propolis; the bees also place propolis in the nest (Figure S1) as a sanitary barrier to prevent microbial infections [8]. *M. ferruginea* use cerumen, a mixture of wax and plant resins, to build protective and supporting nest structures as well as honey pots (Figure S1). The chemistry of these materials has never been studied. The sugar content of *M. ferruginea* honey was found to be from 9.1 to 63.4% [9], and it has demonstrated antimicrobial properties [10], but no individual sugars or other constituents have been identified.

In our study, one honey, two cerumen, and two resin deposits (propolis) samples were studied. This small number was an obvious limitation to the soundness of possible conclusions. However, our findings, being among the first concerning African stingless bees, demonstrated the importance of studies of the products of African Meliponini. The honey chemical composition was studied by ^1^H and ^13^C NMR, which had been successfully applied in research of honey samples, including stingless bee honey [11,12]. Multivariate analysis (PCA—principal component analysis and HCA—hierarchical clustering) and Nightingale’s diagram were performed based on the 13C-NMR data applying Excel and Simca15 [12,13,14]. Propolis and cerumen were analyzed by GC-MS of silylated ethanol extracts. The antimicrobial properties of propolis and cerumen were also studied.

## 2. Materials and Methods

### 2.1. Sample Collection

Propolis, cerumen, and pot-honey were collected in late November 2018 on the west slope of Kilimanjaro, Ngarony locality, Melliponiary of Mr. Baraka Nkini (3°9′8″ S 37°6′54″ E), altitude 1650 m, and stored cold since. Two propolis (samples KC1) and two cerumen (samples KC2) were taken from two different hives, KC1-1 and KC2-1, and KC1-2 and KC2-2, respectively. The pot-honey (TZ) originated from the hive from which the propolis and cerumen samples KC1-1 and KC2-1 originated. The bee species was determined by Dr. Connal Eardley (Plant Protection Research Institute, Pretoria, South Africa); Dr. Alain Pauly (Royal Belgian Institute of Natural Sciences, Bruxelles, Belgium) classified this black form as *Meliponula togoensis*. The origin of Bulgarian honey samples (BG), produced by European honeybees (*A. mellifera*), is described in Table S1. Five polyfloral and five honeydew honeys were selected because they were the two most common Bulgarian honey types.

### 2.2. Honey Sample Preparation

Three hundred twenty milligrams of honey were dissolved in 418 μL distilled water and 187 μL phosphate buffer solution (pH 4.5) containing 0.1% TSP (Na salt of 3-(trimethylsilyl)-2,2,3,3-tetradeuteropropionic acid, Sigma-Aldrich, Darmstadt, Germany) and 0.05% NaN_3_. Small quantities of 0.1M H_3_PO_4_ or 0.1M NaOH were added to the samples for pH adjustment to 4.20.

### 2.3. Honey NMR Spectroscopy

NMR spectra were recorded immediately after sample preparation on a Bruker Avance II+ 600 spectrometer (Biospin GmbH, Rheinstetten, Germany) at 300.0 ± 0.1 K. 1D ^1^H and ^13^C NMR spectra were used for chemical profiling, while various 2D (JRES, COSY, TOCSY, HSQC, and HMBC) NMR spectra provided an unambiguous identification of a number of components in the honeys. Standard Bruker pulse sequences noesypr1d and zgdc30 were used for the acquisition of proton spectra with water suppression and broadband-decoupled carbon spectra. The ^1^H/^13^C NMR spectra were collected using a 30° pulse with 10.6/238 ppm spectral width, 64 K data points, 256/8 K scans, 16 dummy scans, acquisition time of 5.15/0.90 s, relaxation delay of 2.00/1.05 s, composite pulse decoupling with bi_waltz16_32. The chemical shift scale was referenced internally to the TSP ^1^H signal/anomeric ^13^C NMR signal of α-fructofuranose at 0.00 ppm/104.34 ppm.

For experimental details of the quantification of honey constituents, see Appendix A.

### 2.4. Extraction of Propolis and Cerumen. Sample Preparation

Propolis and cerumen samples, grated after cooling, were extracted with 70% ethanol (1:10, *w*/*v*) at room temperature (2 × 24 h). After evaporation in vacuo, the dry extracts were silylated (about 5 mg dry extract was mixed with 50 µL of dry pyridine and 75 µL of N,O-bis(trimethylsilyl)trifluoracetamide (Sigma-Aldrich, Darmstadt, Germany), followed by heating at 80 °C for 20 min) and subjected to GC-MS analysis.

### 2.5. GC-MS Analysis

The GC-MS analysis was performed with Hewlett-Packard 5890 series II Plus, linked to a Hewlett–Packard 5972 mass spectrometer system equipped with a 30 m DB-17HT capillary column, 0.25 mm i.d., 0.15 µm film thickness. The temperature program from 100 to 320 °C at a rate of 5 °C/min; carrier gas Helium at a flow rate of 0.8 mL/min. The split ratio was 75:1, the injector temperature 300 °C, and the ionization voltage 70 eV. The compounds identification was accomplished using commercial libraries, literature data, and/or comparison with mass spectra of reference compounds.

### 2.6. Biological Tests

One propolis and one cerumen sample, KC1-1 and KC2-1, both from the same hive, were subjected to biological tests because the amounts of the other two samples were too small.

#### 2.6.1. Microbial Strains

Staphylococcus aureus ATCC 25923, Enterococcus faecalis ATCC 29212, Listeria monocytogenes ATCC 7644, Pseudomonas aeruginosa ATCC 27853, Salmonella typhi ATCC 14028, Escherichia coli ATCC 25922, Candida albicans ATCC 10239 were used in the study.

#### 2.6.2. Determination of Minimal Inhibitory Concentration (MIC)

MICs were determined by a microtiter broth dilution method [15]. MIC is the lowest extract concentration necessary to inhibit visible growth. The test medium was a Mueller–Hinton broth; the density of the bacteria was 5 × 10^5^ colony-forming units (CFU)/mL. Cell suspensions (100 μL) were inoculated into the wells of 96-well microtiter plates in the presence of extracts of the samples KC1-1 and KC2-1 with different concentrations (5, 2.5, 1.25, 0.625, 0.312, 0.1563, 0.0782 mg dry extract/mL) and incubated at 37 °C for 24 h.

#### 2.6.3. Effect of Extract on Bacterial Biofilm Formation

The effect of KC1-1 and KC2-1 extracts at 1, 1/2, 1/4, 1/8, and 1/16 MIC on the biofilm-forming ability of 7 microorganisms was tested by microplate biofilm assay [16]. 1% of overnight cultures of isolates were added to 200 μL of fresh Tryptose-Soy Broth (TSB) supplemented with 0.25% glucose and cultivated in presence and absence of the test sample for 48 h at 37 °C. Wells containing TSB+ cells served as control. After incubation, the wells were washed with water, and the remaining bacteria were stained with a 0.1% crystal violet solution for 10 min, washed to remove the crystal violet solution, and 200 μL of 33% glacial acetic acid were poured. After shaking, 125 μL of the solution from each well were transferred to a sterile tube, and the volume was adjusted to 1 mL with distilled water. The optical density (OD) of each well was measured at 550 nm (Thermo Scientific Multiskan FC, Vantaa, Finland). Percentage of inhibition was calculated:

(1) $$\mathrm{Biofilm}\;\mathrm{inhibition}\;(\%)=\;\;\frac{\mathrm{OD}550_{Control}-\mathrm{OD}550_{Sample}}{\mathrm{OD}550_{Control}}\;\times \;100$$

#### 2.6.4. Quorum Sensing Inhibition (QSI) Activity on *Chromobacterium violaceum* CV026

QSI was evaluated as described elsewhere [15] with slight modifications. Five milliliters of warm molten Soft Top Agar (1.3 g agar, 2.0 g tryptone, 1.0 g sodium chloride, 200 mL deionized water) was seeded with 100 µL of an overnight *C. violaceum* CV026 culture, and 20 µL of 100 µg/mL hexanoyl homoserine lactone (C6HSL) was added as exogenous acyl-homoserine lactone (AHL) source. Wells of 5 mm diameter were made on each plate after the overlay solidified. Each well was filled with 50 µL of MIC and sub-MIC concentrations (MIC to MIC/8) of the extracts of KC1-1 and KC2-1. A white or cream-colored halo around this well against a purple lawn of activated CV026 bacteria was an indicator of QSI. A clear halo indicated antimicrobial (AM) activity. Each experiment was done in triplicate, and plates were incubated at 30 °C for 3 days, after which the diameters of the QSI zones were measured.

#### 2.6.5. Violacein Inhibition Assay on *C. violaceum* CV12472

The samples were subjected to qualitative analysis to find their QSI potentials against *C. violaceum* CV12472 [17]. Overnight culture (10 µL) of *C. violaceum* (adjusted to 0.4 OD at 600 nm) was added into the sterile microtiter plates containing 200 µL of LB broth and incubated in the presence and absence of MIC and sub-MICs of KC1-1 and KC2-1 extracts. LB broth containing *C. violaceum* ATCC 12,472 was a positive control. The microplates were incubated at 30 °C for 24 h and the reduction in violacein production was observed, absorbance was read at 585 nm. The percentage of violacein inhibition was calculated by the formula:

(2) $$\mathrm{Violacein}\;\mathrm{inhibition}\;(\%)=\frac{\mathrm{OD}585_{Control}-\mathrm{OD}585_{Sample}}{\mathrm{OD}585_{Control}}\;\times \;100$$

#### 2.6.6. Swarming and Swimming Motility Inhibition on *P. aeruginosa* PA01

The inhibition of a swarming motility assay was done as described previously [18]. Briefly, overnight cultures of *P. aeruginosa* PAO1 strain were point-inoculated at the center of swarming plates consisting of 1% peptone, 0.5% NaCl, 0.5% agar, and 0.5% of filter-sterilized D-glucose with various concentrations of KC1-1 or KC2-1 (50, 75, and 100 µg/mL), a plate without the samples was used as control. Plates were incubated for 18 h. The swarming migration was recorded by following swarm fronts of the bacterial cells.

For swimming motility assay, the *P. aeruginosa* PAO1 strain was inoculated at the center of the swarming agar medium consisting of 1% peptone, 0.5% NaCl, 1.5% agar, and 0.5% of filter-sterilized D-glucose with increasing concentrations of propolis/cerumen (50, 75 and 100 μg/mL). The plates were then wrapped with Saran Wrap to prevent dehydration and incubated at 37 °C in an upright position. The reduction in swimming migration was recorded by measuring the swim zones of the cells after 16 h.

## 3. Results

### 3.1. Honey Chemical Composition

Chemical profiling of the honey samples was performed by ^1^H and ^13^C NMR spectroscopy. We used ^13^C NMR spectra for carbohydrate profiling and ^1^H NMR for profiling the remaining compounds, most of them available in smaller quantities. The NMR spectra of *M. ferruginea* honey were measured twice: immediately after receiving it (TZ_1) and again after it was stored for 18 months (TZ_18).

**Figure 1:**
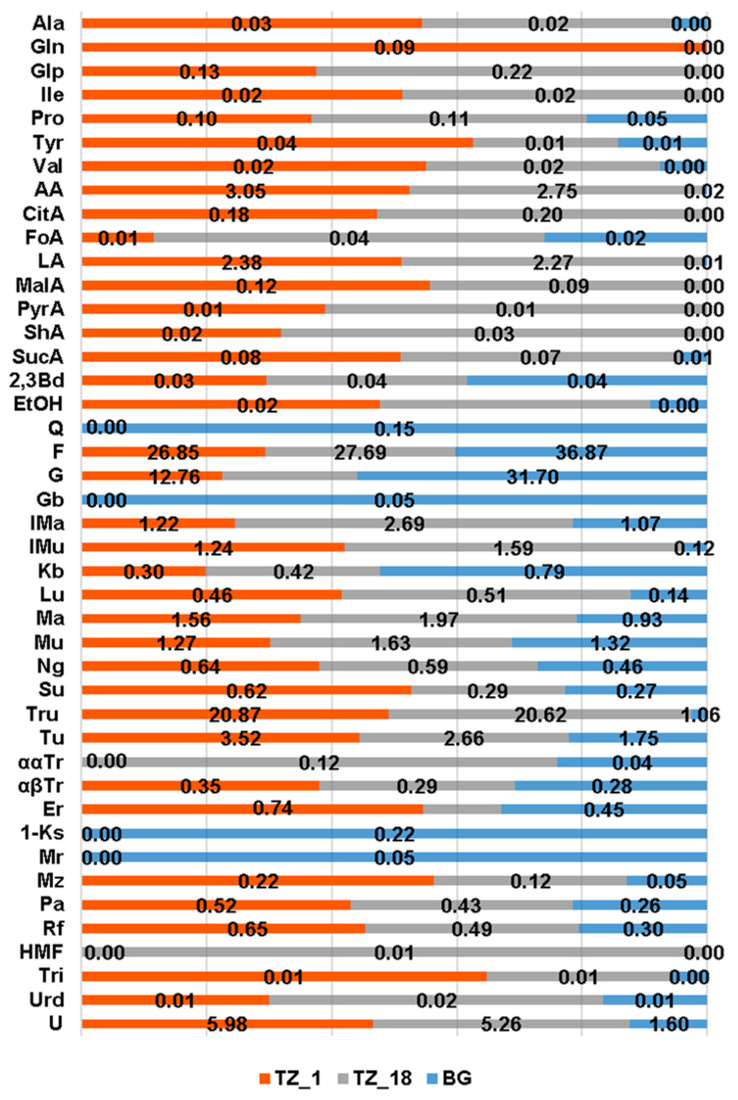
Quantities of honey components (in g/100 g) in *M. ferruginea* honey (TZ_1), *M. ferruginea* honey after 18 months of storage (TZ_18), and Bulgarian honey (BG) (average values of 10 samples). Acronyms: Ala—Alanine; Gln—Glutamine; Glp—Pyroglutamic acid; Ile—Isoleucine; Pro—Proline; Tyr—Tyrosine; Val—Valine; AA—Acetic acid; CitA—Citric acid; FoA—Formic acid; LA—Lactic acid; MalA—Malic acid; PyrA—Pyruvic acid; ShA—Shikimic acid; SucA—Succinic acid; 2,3Bd—2,3 Butanediol; EtOH—Ethanol; Q—Quercitol; F—Fructose; G—Glucose; Gb—Gentiobiose; Ima—Isomaltose; IMu—Isomaltulose; Kb—Kojibiose; Lu—Leucrose; Ma—Maltose; Mu—Maltulose; Ng—Nigerose; Su—Sucrose; Tru—Trehalulose; Tu—Turanose; ααTr—ααTrehalose; αβTr—αβTrehalose; Er—Erlose; 1-Ks—1-Kestose; Mr—Maltotriose; Mz—Melezitose; Pa—Panose; Rf—Raffinose; HMF—5-Hydroxymethylfurfural; Tri—Trigonelline; Urd—Uridine; U—Unknown.

The results were compared to a chemical profile based on 10 typical Bulgarian honeys of *A. mellifera* in order to stress the specificity of the stingless bee honey. We also traced its changes during storage that occurred to be quite different from *A. mellifera* honeys which do not change appreciably for at least 2 years [19]. The specific profiles are presented in Figure 1, indicating concentrations of the studied compounds, and illustrate the differences and changes in the quantities of a number of components quantifiable by NMR: sugars, amino and organic acids, alcohols, nucleobases and hydroxymethylfurfural (HMF). Additionally, the quantities of the sugars determined, together with the chemical shifts of the signals used for quantitation, are presented in Table S2.

### 3.2. Propolis/Cerumen Chemical Composition

The silylated ethanol extracts of *M. ferruginea* propolis and cerumen were analyzed by GC-MS, a technique widely used in chemical profiling of propolis because of the resolving power of capillary GC combined with the valuable structure information provided by EIMS (electon impact mass specvtrometry) [20]. A total of over 50 individual constituents were positively or tentatively identified; for this reason, the chemical profiles are presented in Table 1 by the main chemical classes of compounds and their abundances. The data of individual constituents are given in Table S3. All the samples differed noticeably in their chemistry. The cerumen samples displayed high amounts of sugars and triterpenes; KC2-2 contained also some diterpenes. Two significant components of KC1-1 remained unidentified.

### 3.3. Propolis/Cerumen Antimicrobial Activity

#### 3.3.1. Determination of MIC

The MIC values of the extracts of KC1-1 and KC2-1 against the studied 7 microorganisms are presented in Table 2. They had MICs from 0.1563 to 2.5 mg/mL against the tested strains.

#### 3.3.2. Inhibitory Potential against Violacein Synthesis

Experiments were conducted using *C. violaceum* CV12472 with sub-MICs (from MIC to MIC/256). Data are displayed in Table 3. Complete lack of inhibition was observed only at MIC/256 for KC1-1 and for MIC/128 and MIC/256 for KC2-1.

#### 3.3.3. Antibiofilm Activity

**Figure 2:**
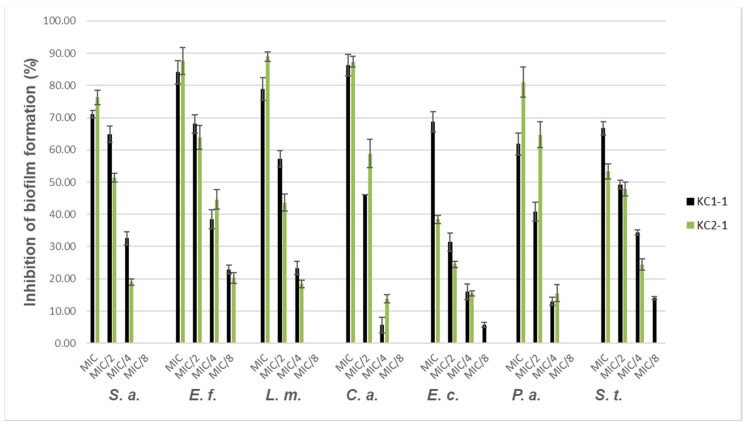
Antibiofilm activity of propolis and cerumen extracts. Note: *S. a.* (*S. aureus* ATCC 25923); *E. f.* (*E. faecalis* ATCC 29212); *L. m.* (*L. monocytogenes* ATCC 7644); *C. a.* (*C. albicans* ATCC 10239); *E. c.* (*E. coli* ATCC 25922); *P. a.* (*P. aeruginosa* ATCC 27853); *S. t.* (*S. typhi* ATCC 14028).

The percentage inhibition of the biofilm formation of the tested microorganisms for several sub-MICs is represented in Figure 2. Levels of suppression of biofilms of 53.32 ± 2.40% to 88.97 ± 1.51% were obtained at MIC for both samples. The only exclusion was KC2-1 with *E. coli* (38% of inhibition). No inhibition of biofilm formation was observed at MIC/16 for the two samples for all microorganisms tested.

#### 3.3.4. Quorum Sensing Inhibition (QSI) Activity on C. Violaceum CV026

MIC and sub-MICs (MIC/2, MIC/4, MIC/8) were tested. The zones of inhibition observed are presented in Table 4. For KC2-1, only the MIC demonstrated inhibition, while KC1-1 inhibited QS also at MIC/2, and MIC/4, MIC/8 was inactive.

#### 3.3.5. Inhibition Assay of Swarming and Swimming Motility

The studied samples exhibited an inhibitory effect against the motility of *P. aeruginosa* PA01 at sub-MIC (100, 75, and 50 µg/mL)–Table 5.

## 4. Discussion

### 4.1. Honey Chemical Profile

Stingless bees’ honey is widely used in traditional medicine in tropical regions to treat bruises, wounds, tumors, ocular cataracts, inflammation, infections, varicose veins, kidney diseases, etc. [11]. Contemporary science has found that most traditional uses have great potential as an added value in modern medicine and has considered this honey to have a higher medicinal value than *A. mellifera* honey [20]. Meliponini honey, also called pot-honey, has organoleptic and physicochemical characteristics, which are completely different from that produced by *A. mellifera* [21,22]. This is due to the different technology used by the bees: *A. mellifera* remove moisture by using their wings and add enzymes in order to digest sugars and conserve honey. On the other hand, stingless bees dehydrate honey to a specific level [23], and after being stored, microorganisms, mainly bacteria and yeasts, will consume part of the sugars and transform them by fermentation processes [24,25] into ethanol, acetic acid, and lactic acid. Thus, the honey of meliponines is characterized by higher acidity and higher water content than the honey of *A. mellifera* [26].

The chemical composition of stingless bee honey is much less studied than that of *A. mellifera* honey for obvious reasons: it has been used by local people for centuries, but it is generally not recognized by the current food standards as honey, and the amounts marketed are much lower. The composition of pot-honey depends on both the nectar sources and bee species. Studies on African Meliponini honey are particularly scarce; existing studies are limited to the quantitative determination of its phenolic and flavonoid compounds [23]. So even though we had access to only one sample, it was of interest to analyze the chemical profile of *M. ferruginea* honey that has not been studied so far, NMR Spectroscopy is one of the most suitable methods for chemical profiling of a number of organic ingredients in honey, giving a very characteristic pattern with the option to quantify simultaneously mono-, di-, trisaccharides, amino and organic acids, nucleobases, HMF, and other characteristic constituents. The comparison of fresh and stored pot-honey was not only of interest with respect to the shelf life of this valuable product [10,27], but it turned out to be worthy of a more detailed study since the stingless honey stability was quite different from that of *A. mellifera*. As a reference, mean values of typical Bulgarian *A. mellifera* honeys were used; their chemical profile was typical for European honeys and did not change considerably for at least two years.

**Figure 3:**
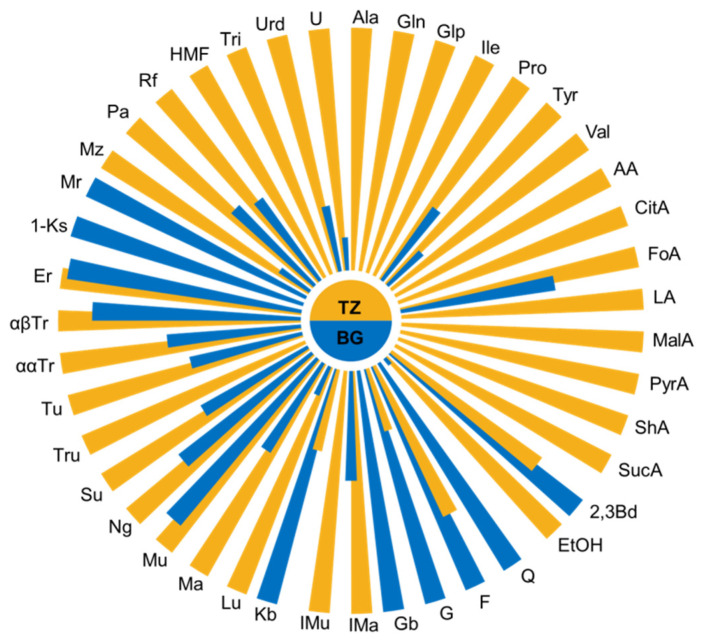
Nightingale’s diagrams for the average content of studied components in *M. ferruginea* (TZ) and Bulgarian *A. mellifera* honeys (BG). Acronyms according to Figure 1.

The most striking difference in the chemical profile of *A. mellifera* and *M. ferruginea* honey was the presence of the unusual disaccharide trehalulose (Tru) as a major component representing more than 20 g/100 g in both fresh and stored stingless bees’ honey (Figure 3 and Table S2).

**Figure 4:**
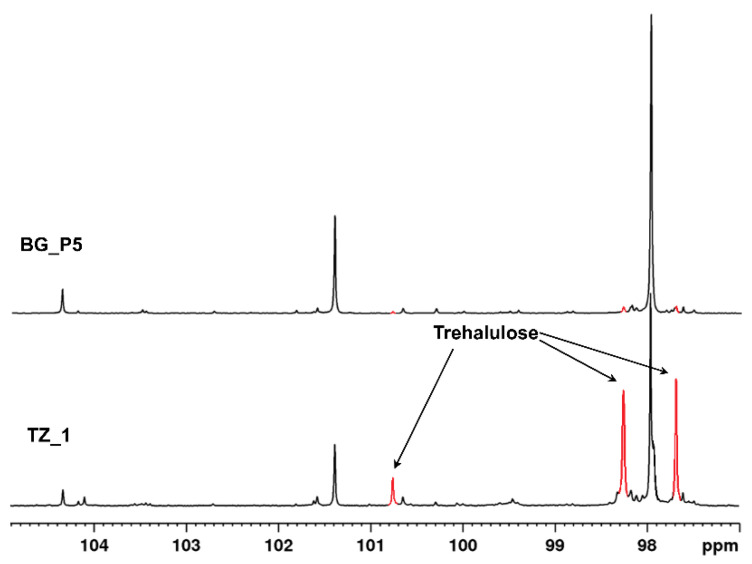
Anomeric ^13^C NMR signals in the region 96–105 ppm of *M. ferruginea* honey (TZ_1) and *A. mellifera* honey (BG_P5) with anomeric signals of trehalulose indicated.

The anomeric signals of trehalulose could be easily detected in the ^13^C NMR spectra of pot-honey (Figure 4). Trehalulose was an unusual α-(1 → 1) glucose-fructose isomer of sucrose with known acariogenic [28] and low glycemic index properties [29]. This disaccharide was recently detected for the first time in Meliponini honeys from different locations (Australia, Malaysia, and Brazil), and five different bee species were detected by Fletcher et al. [30], who suggested that trehalulose could be a marker for the authenticity of pot-honey. Its presence in the studied African stingless bee honey confirmed that it could be a biomarker for stingless bee honey. It was possible that this disaccharide was the result of some enzymatic processes and did not come from the floral sources of the nectar [31].

It should be noted that the monosaccharide content in *M. ferruginea* honey was less than 40%, and the fructose/glucose ratio was above 2, which has not been reported so far in any other honey type. Pot-honey contained more di- and trisaccharides, probably also due to different enzyme activity. These findings indicated that stingless bee honey quality standards, as far as existing, deserved new attention in order to contribute to consumer confidence and prevent adulteration.

Amino and organic acids were more abundant in *M. ferruginea* than in *A. mellifera* honey, as illustrated in Figure 3 and Table S2. The higher values of total acidity and moisture content in stingless bee honey were in line with the studies so far [25]. As already reported, the acid profile comprised high quantities of both acetic (3.05 g/100 g) and lactic (2.38 g/100 g) acids. The major amino acid, except proline in *M. ferruginea* honey, was pyroglutamic acid, rarely found in European honey. In general, both amino- and organic acids were in somewhat higher quantities in pot-honey than in *A. mellifera* honey. The content of ethanol was also higher in *M. ferruginea* honey, as were the nucleobases trigonelline and uridine.

**Figure 5:**
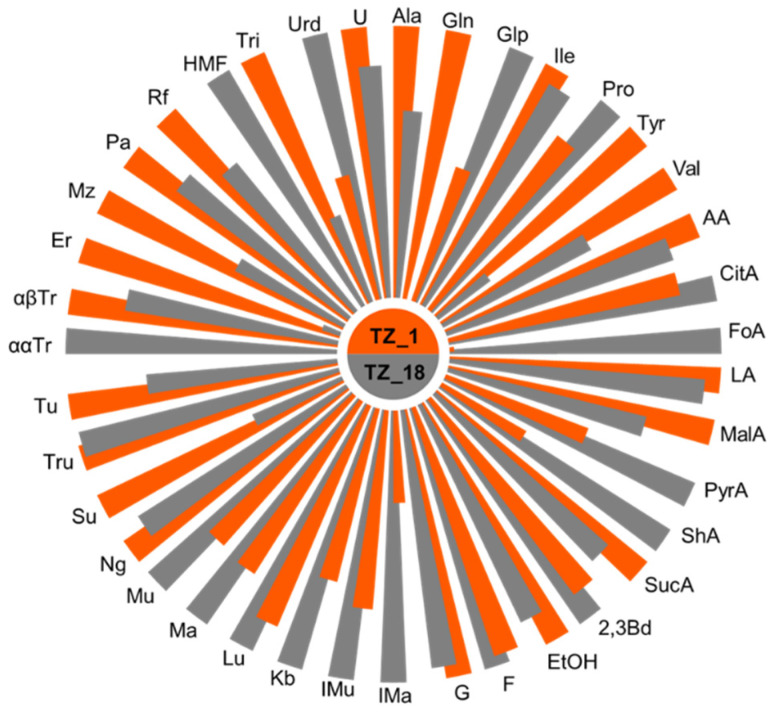
Nightingale’s diagrams illustrating the change in the quantities of the components studied in *M. ferruginea* honey after 18 months (TZ_1 vs. TZ_18). Acronyms according to Figure 1.

Quite remarkable was the change in the chemical profile of stingless bee honey over time, unlike *A. mellifera* honey. Figure 5 and Table S2 showed the changes in the amounts of the studied constituents after 18 months, indicating that storage at room temperature did not prevent various chemical and biochemical processes [27].

Interestingly, the stored pot-honey had higher concentrations of fructose, probably due to degradation of some fructose-containing di- and trisaccharides, while in most European honeys, monosaccharides were converted into disaccharides after storage [32]. Other changes in the carbohydrate profile can be observed in Figure 5. Detected was an increase in the quantity of pyroglutamic acid (from 0.13 g to 0.22 g/100 g), probably due to microbiological activity and Maillard reactions [33]. The amount of other amino acids decreased over storage time, while proline increased, and isoleucine remained stable.

Differences in organic acid contents between fresh and stored honey were visible (Figure 5 and Table S2), while small differences in the content of 2,3-butanediol and ethanol were also detected. As in *A. mellifera* honey, HMF concentration increased with time [34].

**Figure 6:**
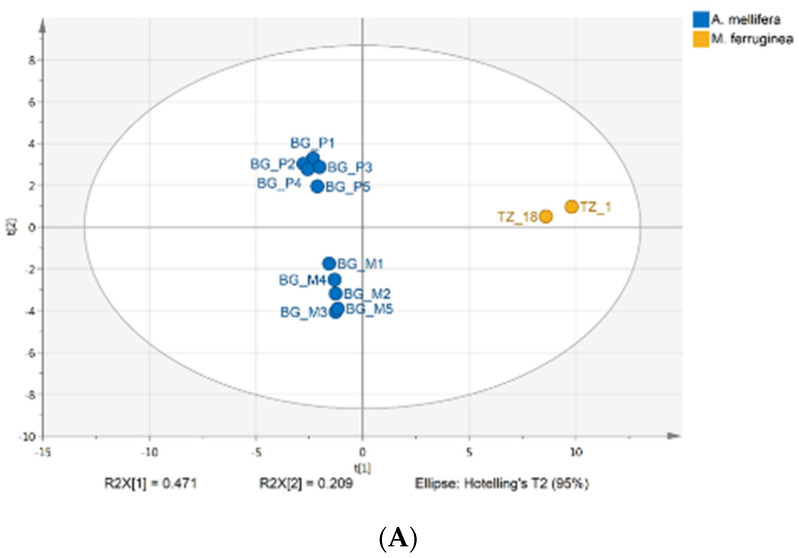

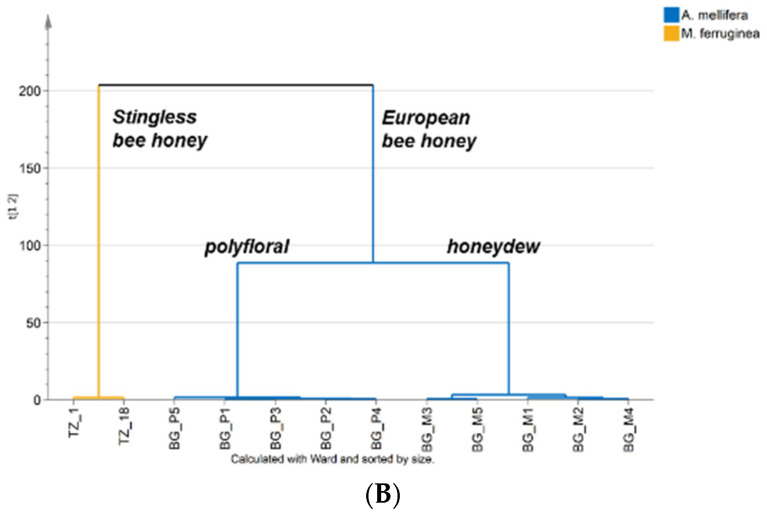
(**A**). Principal component analysis (PCA) score plot of honey samples based on chemical composition. (**B**). Dendrogram of honey samples constructed in chemical composition.

The difference between *M. ferruginea* and *A. mellifera* honey was also demonstrated by PCA and hierarchical cluster analysis based on the NMR quantification of honey constituents (Figure 6A,B).

Bulgarian samples according to botanical origin—polyfloral and honeydew—formed two distinct groups, but the pot-honey (fresh and stored) was clearly separated from both of them.

### 4.2. Propolis/Cerumen Chemical Profiles

Propolis of stingless bees has been attracting the attention of researchers for the last decade because of its remarkable pharmacological properties. A significant number of articles have been published, dedicated to bee species of the Americas, Asia, and Australia (recent reviews: [2,4]), but there is almost nothing known about stingless bees’ propolis from Africa. Only two such articles have been published so far. They report on antimicrobial properties of propolis of *Dactylurina schimidti* from Kenya [35] and *D. studingeri* from Nigeria [36] and contain no chemical information.

The so far published chemical data revealed an enormous chemical diversity of the resins used for nest construction and defense by stingless bees in general, even for representatives of the same species [20]. Our present results showed dissimilarity for propolis and cerumen of *M. ferruginea* at the same location, just from different hives. KC1-1 was rich in diterpenic acids of labdane and abietane type, characteristic for conifers. In the area, there were plantations with *Pinus patula*, *P. radiata*, and *Cupressus lusitanica* [37], which could be resin sources. However, the lack in KC1-1 of detectable amounts of totarol and ferruginol, typical markers of the Cupressaceae family [38] suggested that *Pinus* species were the likely propolis source. In addition, this sample contained two compounds, which could not be identified even tentatively (Table S3). Thus, the plant origin of this sample could be mixed, but the second source remains unknown.

In the sample KC1-2, unlike KC1-1, no diterpenes could be detected. Its major constituents were triterpenes: mainly alcohols, acetates, and ketones with oleanan/ursan and lupan skeleton. These triterpenes are often found in stingless bees propolis, as well as in *A. mellifera* tropical propolis. They are usual components of many tropical plant species and their botanical origin can hardly be identified. A substantial amount of quinic acid, accompanied by some caffeoylquinic acids, were also identified in this sample. In principle the presence of hydroxycinnamic acids conjugated with quinic acid in resins and in propolis is not rare [39,40,41]. The presence of water soluble quinic acid in resins, however, is unusual. On the other hand, quinic acid has been found in the latex of some tropical plants [42,43]. Its origin in our sample is yet unknown.

In addition, KC1-2 contained alkylphenols/resorcinols, anacardic acids, and mangiferolic acid, most probably originating from *Mangifera indica* fruit bark [42]. They have been detected in propolis of stingless bee species in Asia and South America [44], as well as in tropical propolis of *A. mellifera* [45,46,47,48]. It is known that, in many cases, the propolis botanical sources used by *A. mellifera* and stingless bees did not coincide, but mango tree was one of the important exceptions.

The two cerumen were also somewhat different from one another. Both of them contained substantial amounts of carbohydrates and triterpenes. Their triterpenic fingerprints were similar; they were also very close to the triterpene composition of KC1-2, possibly all of them shared a common resin source. The compositions of the sugar parts were dissimilar. In KC2-1, it consisted mainly of arabitol and mannitol and small amounts of monosaccharides, while these sugar alcohols were only minor components of KC2-2. It is interesting to note that arabitol and mannitol are common storage substances in fungal spores [49]. It is known that various species of molds and yeasts occur in nests and bee guts of Meliponini, they are supposed to play a role in honey maturation [50], and the presence of the two alcohols could be related to the high concentration of their spores in the respective nest. While KC2-1 contained no detectable amounts of diterpenes, the diterpenes in KC2-2 were almost the same as these of KC1-1.

In general, the four studied samples had qualitative and quantitative differences in their chemistry. This fact was an indication that *M. ferruginea* had no strict preferences for resins used to construct and protect their nests.

### 4.3. Propolis/Cerumen Antimicrobial and Anti-Quorum Sensing Activity

The emergence of multidrug-resistant microbial pathogens, usually resulting from misuse of conventional antibiotics, has caused an elapse in their efficacy, and researchers are turning towards the search for novel antimicrobial agents [17,51]. It is very important to search for new therapies, and an appropriate strategy seems to be the disruption of bacterial cell-to-cell communication networks, known as quorum sensing (QS), the inhibition of microbial biofilms as well as motilities [52,53]. Propolis is a safe and natural antimicrobial, which could be an alternative to antibiotics. However, most studies reported only its inhibitory and bactericidal effects without examining their effects on QS-mediated traits in pathogenic bacteria.

For this reason, the antimicrobial properties of propolis and cerumen of *M. ferruginea* were tested. We studied not only MIC but also the anti-QS potential of the extracts. The investigation of QS systems could provide us with powerful tools against harmful bacteria [54]. Only two samples were subjected to the tests—one propolis KC1-1 and one cerumen KC2-1—because the available amounts of the other two were insufficient for the experiments.

#### 4.3.1. Determination of MIC

The MICs against the studied Gram-positive and Gram-negative bacteria were in the range of values found for propolis (Table 2). Their activity against most microorganisms tested might be regarded as moderate (MIC > 2000 µg/mL was classified as inactive [55]). It was important to note that, in general, the MICs of the propolis were lower than the ones of the cerumen (with the exclusion of MICs against *P. aeruginosa* and *S. typhi*). This could be due to the differences in their chemical composition: KC1-1 was rich in diterpenes known to possess antibacterial potential [56], while KC2-1 contained no diterpenes. Velikova et al. [57] had found that in the propolis of the Brazilian Meliponini, high antibacterial activity was related to a high percentage of diterpenic acids.

#### 4.3.2. Inhibitory Potential against Violacein Synthesis

Experiments were conducted with sub-MICs (MIC/2 to MIC/256). The inhibition of violacein synthesis was an indicator of the inhibition of a chemical signaling process, which was mediated by AHLs, mediators of QS [56]. Complete inhibition of violacein production was evidenced at MIC/8 for KC1-1 and MIC/4 for KC2-1. A concentration-dependent inhibition was observed with both extracts. In this case, too, the propolis sample rich in diterpenes was more active. It should be mentioned that the two unidentified constituents present in substantial amounts in this sample (see Table 1), could also contribute to this activity.

#### 4.3.3. Antibiofilm Activity

Biofilm increases the resistance of bacteria to antimicrobial agents, thus being able to act as a persistent source of pathogenic bacteria. At present, there are no therapies that effectively target microbial biofilms, which are intrinsically resistant to conventional antibiotics [58]. For this reason, the search for innovative biofilm inhibitors has been attracting growing attention [59]. The extracts of propolis and cerumen-inhibited biofilm formation by all microorganisms were tested at MIC, MIC/2, and MIC/4 (Figure 2). The concentration had a direct relationship with the inhibitory effect. For both samples, the highest inhibition was observed at MIC, and MIC/16 had no effect for all microorganisms studied. The inhibition of the biofilm formation by propolis and cerumen was different towards different bacteria. The highest activity was observed with KC2-1 against *L. monocytogenes*-89% inhibition, and *E. faecalis* 88% inhibition (0.625 mg/mL), and with KC1-1 against *L. monocytogenes*—79% inhibition at 0.1563 mg/mL. In this case, a correlation of chemical composition and activity was hard to establish. The values observed were comparable with the results obtained with plant extracts: 80–90% inhibition against *L. monocytogenes* [60].

#### 4.3.4. Quorum Sensing Inhibition (QSI) Activity on *C. violaceum* CV026

As it is obvious from Table 4, KC1-1 was a much more potent inhibitor of QS. The already mentioned chemical differences between the samples, and especially the presence of significant amounts of diterpenic acids in propolis KC1-1, which were lacking in cerumen KC2-1, could be responsible for this. The high potential to disrupt AHL-dependent QS communication, using the *C. violaceum* CV026, was also demonstrated for propolis from honeybees, *A. mellifera* rich in phenolic compounds [61].

#### 4.3.5. Inhibition Assay of Swarming and Swimming Motility

Virulence and invasion capabilities, as well as antibiotic resistance of some bacteria have been attributed to their swarming [62]. Thus, swarming and swimming motility inhibition could be important. Inhibition of swarming and swimming motility of *P. aeruginosa* PA01 was higher for the cerumen extract compared to the propolis extract (Table 5). The presence of triterpenes in KC2-1 could be of some importance in this case. Recently, pentacyclic triterpenes were found to significantly reduce swarming and swimming motility of *P. aeruginosa* [63].

## 5. Conclusions

In conclusion, it was established that *M. ferruginea* collected resins from a number of plants available in the vicinity of the hives and did not have one preferred resin source. This could result in propolis and cerumen with highly variable chemical compositions. The honey of these stingless bees contained high amounts of the disaccharide trehalulose, which might be responsible for some of the reported biological activities of stingless bee honey and was rich in organic acids. Our results, although preliminary and based on a limited number of samples, demonstrated that the honey, cerumen, and propolis of African stingless bees are rich in biologically active substances and deserve further research. The results of such research could lead to increased use, demand, and prices for Meliponini products and provide an additional source of income for farmers in rural communities.

## Figures and Tables

**Table 1:** Chemical composition of propolis and cerumen extracts by compound class (GC-MS; % of TIC (Total Ion Current).

Compound Class	KC1-1	KC1-2	KC2-1	KC2-2
Sugars and sugar derivatives	15.6	9.1	35.1	54.1
Aromatic acids	-	1.8	0.2	-
Fatty acids	2.0	4.6	3.6	0.9
Diterpenes (acids)	60.6	-	-	11.9
Cardanol C17:1	-	0.1	-	-
Resorcinols	-	4.1	-	0.2
Anacardic acids	-	2.8	-	0.3
Quinic acid	0.4	15.5	1.2	2.1
Caffeoylquinic acids	-	7.6	-	-
Triterpenes	0.7	37.3	24.4	26.7
Unknown M^+^ = 570	3.9	-	-	-
Unknown M^+^ = 568	8.0	-	-	-

- not detected.

**Table 2:** Antimicrobial activity, minimal inhibitory concentration (MIC; mg/mL).

Micro-Organism	KC 1-1	KC 2-1
*S. aureus* ATCC 25923	0.625	1.25
*E. faecalis* ATCC 29212	0.3125	0.625
*L. monocytogenes* ATCC 7644	0.1563	0.625
*C. albicans* ATCC 10239	0.625	1.25
*E. coli* ATCC 25922	0.625	1.25
*P. aeruginosa* ATCC 27853	0.3125	0.1563
*S. typhi* ATCC 14028	2.5	1.25

**Table 3:** Inhibition of violacein formation (%) using *C. violaceum* CV12472.

Concentration	KC1-1		KC2-1	
	MIC (mg/mL)	MBC (mg/mL)	MIC (mg/mL)	MBC (mg/mL)
	0.625	2.5	2.5	>5
	Violacein Inhibition (%)			
MIC	100 ± 0.0		100 ± 0.0	
MIC/2	100 ± 0.0		100 ± 0.0	
MIC/4	100 ± 0.0		100 ± 0.0	
MIC/8	100 ± 0.0		89.6 ± 0.3	
MIC/16	61.4 ± 5.2		50.1 ± 2.0	
MIC/32	44.2 ± 1.5		28.9 ± 0.4	
MIC/64	21.6 ± 1.1		13.0 ± 0.5	
MIC/128	11.8 ± 2.4		-	
MIC/256	-		-	

- no visible inhibition.

**Table 4:** Anti-quorum sensing activity zones (mm) on *C. violaceum* CV026.

Concentration	KC1-1 (MIC = 2.5 mg/mL)	KC2-1 (MIC = 5 mg/mL)
MIC	16.5 ± 2.3	10.5 ± 0.5
MIC/2	13.8 ± 1.5	-
MIC/4	10.0 ± 4.5	-
MIC/8	-	-

**Table 5:** Swarming and swimming motility inhibition of P. aeruginosa PA01 (%).

Concentration (µg/mL)	KC1-1		KC2-1	
	Swarming	Swimming	Swarming	Swimming
100	42.13 ± 1.75	27.32 ± 2.11	57.14 ± 0.50	29.28 ± 4.18
75	20.70 ± 4.21	6.77 ± 1.00	42.86 ± 5.10	14.27 ± 2.50
50	6.43 ± 0.50	-	20.70 ± 1.00	-

## Data Availability

The dataset of the current study is available from the corresponding authors on reasonable request.

## Supplementary Materials

The following are available online at https://www.mdpi.com/article/10.3390/foods10050997/s1, Supplementary file 1: Figure S1. Hive of *M. ferruginea.* **A**–propolis (resin load), **B**–cerumen; Supplementary file 2: Table S1. Bulgarian honey samples; Supplementary file 3: Table S2. Quantities of honey components (in g/100g) in *M. ferruginea* honey (TZ_1), *M. ferruginea* honey after 18 months of storage (TZ_18), and Bulgarian honey samples (BG_P1–BG_P5, BG_M1–BG_M5). Supplementary file 4: Table S3. Chemical composition of propolis and cerumen extracts (GC-MS; % of TIC).

## Appendix A

Experimental details on the quantification of honey constituents. All 1D ^1^H NMR spectra were subjected to deconvolution using the Quantitative Global Spectral Deconvolution Peak Picking algorithm and manual Line Fitting (setting the Lorentzian and Gaussian character of all peaks to 1) of MestreNova 12.0.1. program (https://mestrelab.com/software/mnova/ accessed on 14 January 2019). By ^1^H NMR spectra, the content of amino and organic acids, alcohols, nucleobases, and HMF was analyzed. For the quantification, the integral areas under the peaks of the identified compounds were measured, and the quantity of the corresponding components (in g/100 g) was determined using the following general formula [64].

m_x_ = N_TSP_/N_x_ ∗ Int_x_/Int_TSP_ ∗ MW_x_/MW_TSP_ ∗ m_TSP_    or (A1)

m_x_ = 9.77 ∗ 10^−4^ ∗ Int_x_/Int_TSP_ ∗ MW_x_/N_x_ (A2)

where N_TSP_ is the number of TSP protons (9); N_x_–number of chemically equivalent protons of the studied compound X, used for quantitation (3 for CH_3_, 2 for CH_2_, 1 for CH, etc.); Int_TSP_–integral area under the peak of TSP; Int_X_–integral area under the peak of compound X used for quantitation; MW_TSP_–molar weight of TSP (172.27 g/mol); MW_X_–molar weight of the studied compound X; m_TSP_–quantity of TSP in the sample measured (0.0187 g/100 g)

The quantities of the various mono-, di-, and trisaccharides were determined from the intensities of the anomeric protons in the ^13^C NMR spectra according to [12].
