# Supplementary material for: A Preliminary Study of Chemical Profiles of Honey, Cerumen, and Propolis of the African Stingless Bee Meliponula ferruginea
Source: Foods. 2021 May 2;10(5):997. doi: 10.3390/foods10050997 (PMC8147412; doi:10.3390/foods10050997)
Supplement: Supplementary file 1 [file foods-10-00997-s001.zip › foods-1215601-supplementary/Table S1.pdf]

Table S1. Bulgarian honey samples

Sample	Supplier	Geographical origin	Botanical origin	Entomological origin
BG_P1	beekeeper	Ihtiman	polyfloral	Apis mellifera
BG_P2	beekeeper	Saedinenie, Plovdiv	polyfloral	Apis mellifera
BG_P3	commercial	*	polyfloral	Apis mellifera
BG_P4	beekeeper	Vratsa	polyfloral	Apis mellifera
BG_P5	beekeeper	Asenovgrad	polyfloral	Apis mellifera
BG_M1	commercial	Stolnik, Sofia	honeydew	Apis mellifera
BG_M2	beekeeper	Kosharevo, Pernik	honeydew	Apis mellifera
BG_M3	beekeeper	Dobrich Province	honeydew	Apis mellifera
BG_M4	beekeeper	Sandanski	honeydew	Apis mellifera
BG_M5	beekeeper	Strandzha	honeydew	Apis mellifera
