# Supplementary material for: A Preliminary Study of Chemical Profiles of Honey, Cerumen, and Propolis of the African Stingless Bee Meliponula ferruginea
Source: Foods. 2021 May 2;10(5):997. doi: 10.3390/foods10050997 (PMC8147412; doi:10.3390/foods10050997)
Supplement: Supplementary file 1 [file foods-10-00997-s001.zip › foods-1215601-supplementary/Table S2-NS_d.pdf]

Table S2. Quantities of honey components (in g/100g) in *M. ferruginea* honey (TZ_1), *M. ferruginea* honey after 18 months' storage (TZ_18) and Bulgarian honey samples (BG_P1 – BG_P5, BG_M1 – BG_M5).

Compound, g/100g	^{13}C NMR chemical shift (δ), ppm	TZ_1	TZ_18	Average (TZ)	Average (BG)	BG_P1	BG_P2	BG_P3	BG_P4	BG_P5	BG_M1	BG_M2	BG_M3	BG_M4	BG_M5	Average (BG_P)	Average (BG_M)
**Monosaccharides**																	
**F**	*67.5*	26.85	27.69	27.27	36.87	35.08	42.23	37.19	37.45	38.64	35.43	35.64	36.64	35.81	34.59	38.12	35.62
**G**	*74.1*	12.76	12.29	12.53	31.70	32.85	27.67	32.35	32.43	31.86	32.85	31.91	30.59	31.34	33.17	31.43	31.97
**Disaccharides**																	
**Gb**	*102.5*	0.00	0.00	0.00	0.05	0.13	0.00	0.00	0.00	0.00	0.00	0.15	0.00	0.17	0.00	0.03	0.06
**IMa**	*97.7*	1.22	2.69	1.96	1.07	0.83	0.76	0.86	0.86	1.22	1.26	1.16	0.83	1.36	1.54	0.91	1.23
**IMu**	*104.7*	1.24	1.59	1.42	0.12	0.00	0.00	0.00	0.00	0.00	0.00	0.00	0.43	0.32	0.46	0.00	0.24
**Kb**	*89.3*	0.30	0.42	0.36	0.79	0.75	0.64	0.82	0.81	0.91	0.76	0.87	0.85	0.93	0.53	0.79	0.79
**Lu**	*100.1*	0.46	0.51	0.49	0.14	0.09	0.11	0.09	0.09	0.11	0.17	0.15	0.15	0.19	0.21	0.10	0.17
**Ma**	*99.5*	1.56	1.97	1.77	0.93	1.72	1.17	1.23	1.44	0.28	0.75	1.03	0.65	0.57	0.48	1.17	0.70
**Mu**	*100.3*	1.27	1.63	1.45	1.32	1.15	1.17	1.00	1.17	1.28	1.39	1.35	1.48	1.34	1.86	1.15	1.48
**Ng**	*98.8*	0.64	0.59	0.62	0.46	0.40	0.41	0.49	0.42	0.56	0.41	0.52	0.55	0.46	0.37	0.46	0.46
**Su**	*76.4*	0.62	0.29	0.46	0.27	0.45	0.23	0.25	0.23	0.19	0.14	0.18	0.69	0.18	0.14	0.27	0.27
**Tru**	*97.7*	20.87	20.62	20.75	1.06	0.81	0.90	0.80	0.86	1.01	1.22	0.98	1.27	1.19	1.55	0.88	1.24
**Tu**	*100.7*	3.52	2.66	3.09	1.75	1.97	1.98	1.64	1.80	1.58	1.81	1.90	1.49	1.92	1.40	1.79	1.70
**ααTr**	*93.0*	0.00	0.12	0.06	0.04	0.05	0.00	0.00	0.00	0.00	0.00	0.05	0.10	0.06	0.12	0.01	0.07
**αβTr**	*102.7*	0.35	0.29	0.32	0.28	0.25	0.26	0.26	0.29	0.29	0.26	0.35	0.33	0.34	0.21	0.27	0.30
**Trisaccharides**																	
**Er**	*99.6*	0.74	0.17	0.46	0.45	1.10	0.74	0.97	0.39	0.26	0.28	0.23	0.20	0.24	0.06	0.69	0.20
**1-Ks**	*92.3*	0.00	0.00	0.00	0.22	0.12	0.00	0.20	0.00	0.00	0.25	0.31	0.49	0.34	0.44	0.06	0.37
**Mr**	*99.3*	0.00	0.00	0.00	0.05	0.00	0.00	0.00	0.00	0.00	0.00	0.23	0.21	0.00	0.03	0.00	0.09
**Mz**	*91.5*	0.22	0.12	0.17	0.05	0.07	0.00	0.00	0.00	0.00	0.12	0.08	0.03	0.21	0.00	0.01	0.09

**Pa**	*99.6*	0.52	0.43	0.48	0.26	0.58	0.24	0.24	0.20	0.27	0.23	0.27	0.25	0.25	0.08	0.31	0.22
**Rf**	*76.2*	0.65	0.49	0.57	0.30	0.27	0.20	0.26	0.23	0.25	0.33	0.33	0.34	0.33	0.42	0.24	0.35
**Other compounds**																	
**Q**	*33.3*	0.00	0.00	0.00	0.15	0.00	0.00	0.00	0.00	0.00	0.28	0.23	0.31	0.36	0.31	0.00	0.30
**U**	-	5.98	5.26	5.62	1.60	1.29	1.27	1.27	1.26	1.24	1.97	1.89	1.97	1.96	1.90	1.27	1.94

Acronyms: F – Fructose; G – Glucose; Gb – Gentiobiose; IMa – Isomaltose; IMu – Isomaltulose; Kb – Kojibiose; Lu – Leucrose; Ma – Maltose; Mu – Maltulose; Ng – Nigerose; Su – Sucrose; Tru – Trehalulose; Tu – Turanose; $\alpha\alpha$Tr – $\alpha\alpha$Trehalose; $\alpha\beta$Tr – $\alpha\beta$Trehalose; Er – Erlose; 1-Ks – 1-Kestose; Mr – Maltotriose; Mz – Melezitose; Pa – Panose; Rf – Raffinose; Q – Quercitol; U – Unknowns (sum of 15 unidentified compounds with signals at δ 104.1 ppm (U1), 103.7 ppm (U2), 103.6 ppm (U3), 103.5 ppm (U4), 103.4 ppm (U5), 103.3 ppm (U6), 102.9 ppm (U7), 102.3 ppm (U8), 101.7 ppm (U9), 101.2 ppm (U10), 100.7 ppm (U11), 98.1 ppm (U12), 97.8 ppm (U13), 96.7 ppm (U14), 12.0 ppm (U15)).
