# Supplementary material for: A Preliminary Study of Chemical Profiles of Honey, Cerumen, and Propolis of the African Stingless Bee Meliponula ferruginea
Source: Foods. 2021 May 2;10(5):997. doi: 10.3390/foods10050997 (PMC8147412; doi:10.3390/foods10050997)
Supplement: Supplementary file 1 [file foods-10-00997-s001.zip › foods-1215601-supplementary/Table S3.pdf]

Table S3. Chemical composition of propolis and cerumen extracts (GC–MS; % of TIC[a]).

**Compound**	**KC1-1**	**KC1-2**	**KC2-1**	**KC2-2**
Sugars (mono- and disaccharides)	15.6	9.1	35.1	54.1
Arabitol	-[b]	-	24.7	1.0
Mannitol	1.6	-	3.8	2.6
p-Hydroxybenzoic acid	-	-	0.2	-
Vanillic acid	-	0.7	-	-
p-Coumaric acid	-	0.6	-	-
Caffeic acid	-	0.5	-	-
Quinic acid	0.4	15.5	1.2	2.1
Hexadecanoic acid	0.3	1.2	1.3	0.2
Hexadecenoic acid	-	0.2	-	-
Oleic acid	1.7	2.0	1.0	0.7
Linoleic acid	-	0.9	-	-
Eicosanoic acid	-	0.3	1.3	-
Diterpenic acid pimaric type	1.3	-	-	-
13-*epi*-Toruloso	0.8	-	-	0.6
Diterpenic acid pimaric type (isomer)	8.8	-	-	2.0
Hydroxy abietadiene	2.1	-	-	
Isopimaric acid	-	-	-	0.2
Communic acid	1.0	-	-	Tr.
13-*epi*-Cupressic acid	3.6	-	-	1.7

Imbricataloic acid	30.79	-	-	5.4
Abietic acid	7.3	-	-	1.5
Dehydroabietic acid	0.1	-	-	0.5
Acetylisocupressic acid	4.8	-	-	Tr.
Unknown M^{+}= 570 (major peaks m/z 453, 439, 218, 147, 129)	3.9	-	-	-
Unknown M^{+}= 568 (major peaks m/z 451, 437, 218, 147, 129)	8.0	-	-	-
Resorcinol C15:0	-	0.3	-	-
Resorcinol C15:1	-	0.3	-	-
Cardanol C17:1	-	0.1	-	--
Resorcinol C17:0	-	0.4	-	-
Resorcinol C17:1	-	0.4	-	-
Resorcinol C17:1 (isomer)	-	2.6	-	0.2
Anacardic acid C15:1	-	0.1	-	-
Resorcinol C19:1	-	0.1	-	-
Anacardic acid C17:1 (isomer)	-	0.5	-	-
Anacardic acid C17:1 (isomer)	-	0.4	-	-
Anacardic acid C17:1 (isomer)	-	1.8	-	0.3
Chlorogenic acid	-	7.6	-	-
Feruloyl-quinic acid	-	Tr.[c]	-	-

4-Caffeoyl-quinic acid	-	Tr.	-	-
5-Caffeoyl-quinic acid	-	Tr.	-	-
Lanosterol	-	0.8	-	1.7
Lanosterol (3-epi)	-	-	0.8	-
Beta-sitosterol	-	0.2	-	-
β-Amyrine	0.2	6.0	6.5	6.8
Cycloartenol	-	3.0	1.2	0.9
α-Amyrine	0.2	6.9	3.1	4.7
Lupeol	0.3	6.8	5.2	6.1
β-Amyrenone	-	3.0	-	1.3
Triterpenic acid	-	-	0.8	-
Oleanolic acid	-	-	-	0.8
α-Amyrenone	-	3.2	-	1.4
β-Amyrine acetate	-	-	2.4	-
Lupenon	-	5.1	-	1.5
Dammarenone	-	-	-	1.5
α-Amyrine acetate	-	0.3	0.9	-
Ketoalcohol betulin type	-	-	3.5	-
Triterpene acetate betulin type	-	0.6	-	-
Mangiferolic acid	-	1.4	-	-

[a] The total ion current generated depends on the characteristics of the compound concerned and is not a true quantitation [b] Not found; [c] Traces (< 0.1)
