# Supplementary figures and images for: A Preliminary Study of Chemical Profiles of Honey, Cerumen, and Propolis of the African Stingless Bee Meliponula ferruginea
Source: Foods. 2021 May 2;10(5):997. doi: 10.3390/foods10050997 (PMC8147412; doi:10.3390/foods10050997)

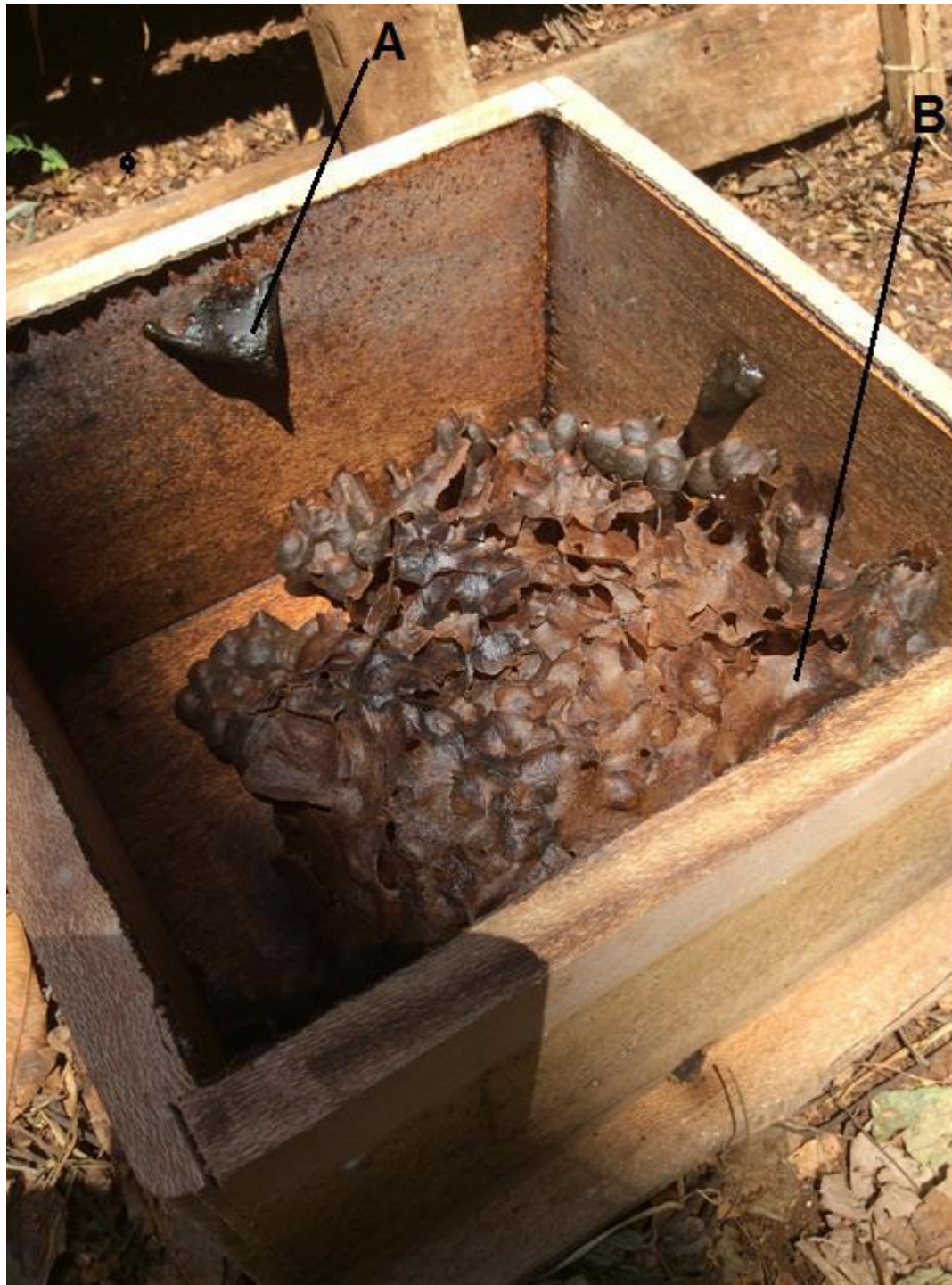

**Fig.S1.** Hive of *M. ferruginea*. **A** – propolis (resin load), **B** – cerumen (Photo: Kerry Clark)

Supplement: Supplementary file 1 [file foods-10-00997-s001.zip › foods-1215601-supplementary/Fig S1..pdf]
